# Effects of High Intensity Interval Training versus Sprint Interval Training on Cardiac Autonomic Modulation in Healthy Women

**DOI:** 10.3390/ijerph191912863

**Published:** 2022-10-08

**Authors:** Jordana Oliveira, Paulo Gentil, João Pedro Naves, Luiz Fernando Souza Filho, Lucas Silva, Antonio Roberto Zamunér, Claudio Andre de Lira, Ana Rebelo

**Affiliations:** 1 Department of Physiotherapy, Araguaia University Center, Goiania 74223-060, Brazil; 2 Faculty of Medicine, Goias Federal University, Goiania 74690-900, Brazil; 3 Faculty of Physical Education and Dance, Goias Federal University, Goiania 74690-900, Brazil; 4 Department of Physiotherapy, Estacio de Sá de Goias University Center, Goiania 74063-010, Brazil; 5 Laboratory of Clinical Research in Kinesiology, Department of Kinesiology, Universidad Católica del Maule, Talca 34809112, Chile; 6 Institute of Biological Sciences, Federal University of Goias, Goiania 74690-900, Brazil

**Keywords:** high-intensity interval training, autonomic nervous system, human physical conditioning

## Abstract

Background: For the prevention of cardiovascular diseases, the practice of physical exercises is an effective strategy in improving or maintaining cardiorespiratory health; however, a lack of time is a barrier to access and interval training appears as possible facilitator. This study aims to compare the effects of two interval training protocols on cardiac autonomic modulation in healthy women. Methods: we conducted a randomized clinical trial with 43 women with a mean age of 29.96 ± 6.25 years, allocated into two groups; high-intensity interval training (HIIT) consisting of four four-minute high-intensity sprints interspersed with three minutes of active recovery and the Sprint interval training (SIT) with four 30-s sprints all-out, interspersed with four minutes of recovery (active or passive). Results: the HIIT group presented better results for the patterns without variation (0V) variables (*p* = 0.022); Shannon entropy (*p* = 0.004) Conditional Entropy (*p* = 0.025). However, there was a significant group effect for some variables, Oxygen Volume (VO2) (*p* = 0.004), Square root of the mean quadratic differences between the adjacent normal R-R intervals (*p* = 0.002) and standard deviation of all normal R-R intervals recorded in a time interval (*p* = 0.003), demonstrating an improvement independent of the protocol. Conclusion: we conclude that eight weeks of interval training were able to produce positive effects on cardiac autonomic modulation in healthy women, with better results for HIIT in this population.

## 1. Introduction

Low cardiorespiratory conditioning and changes in cardiac autonomic modulation evidenced by sympathovagal imbalance are well-known risk factors for the development of cardiovascular and metabolic diseases [1,2]. Therefore, the development of low-cost assessment methods that function as predictors of cardiovascular health is an important alternative for diagnosis and outlining ways to either prevent these diseases or send them into remission [2,3,4].

As alternatives for assessing and stratifying cardiovascular disease risk, heart rate variability (HRV) and heart rate (HR) kinetics are methods that, through cardiac autonomic modulation, assess interdependence between the neural and cardiovascular systems, with the possibility of predicting and alleviating disease progression if associated with the control of modifiable risk factors (SMuRFs) [5,6,7,8].

It is known that the best strategy for controlling SMuRFs is to practice physical exercise and alter unhealthy eating habits [9]. However, a lack of time for self-care remains one of the main barriers preventing adherence to these noninvasive and non-pharmacological approaches [10]. For this purpose, physical exercise programs that use efficient time-type strategies, such as high intensity interval training, emerge as promising and feasible modalities [11,12].

High intensity interval training (HIIT) consists of performing high intensity efforts alternated by periods of passive recovery or low intensity efforts. HIIT has been shown to produce results relating to improved cardiorespiratory fitness, including increased HRV and vagal modulation, training-induced bradycardia, decreased systolic blood pressure, and decreased subcutaneous adiposity and insulin resistance. These results are either similar to or superior to results from continuous training, with the additional benefit of being performed in a shorter time period [13,14,15,16,17,18,19].

However, it is important to note that there is a wide variety of interval training protocols [20], each with different acute and chronic effects [17,18,19], including those relating to autonomic modulation [20]. Two of the most popular HIIT protocols are sprint protocols, which involve maximum efforts of typically 10 to 30 s interspersed with long recovery periods, and long interval training, which involves submaximal efforts of a duration commonly between 2 and 4 min, alternated by intervals of low intensity effort for periods less than or equal to the exertion periods [21].

High intensity performance with lower volumes, in addition to greater popularization and utilization, seems to present the best results in relation to cardiac autonomic modulation; however, the results are still incipient on long-term effects and on HRV and kinetics in relation to exercise and recovery parameters [20,22,23,24,25]. Although there are studies that demonstrate superiority of HIIT for decrease anxiety and depression [18], cardiorespiratory capacity and body composition [16], their effects on autonomic modulation not yet known. Therefore, the aim of this study was to compare the effects of two interval training protocols on HRV responses and HR kinetics in healthy women.

## 2. Materials and Methods

### 2.1. The Participants

Initially, 100 women were recruited via social media and verbal invitation. Inclusion criteria were: (I) being healthy classified as having no disease and (II) physically active in a self-reported manner (~150 min of physical activity per week). Exclusion criteria were: (I) body mass index ≥ 30 kg/m^2^; (II) unstable angina; (III) systemic arterial hypertension; (IV) chronic obstructive pulmonary disease; (V) diabetes mellitus; (VI) neoplasm; (VII) renal failure; (VIII) sequelae of stroke; and (IX) any musculoskeletalchanges that prevented the performance of tests or protocols. Forty-one participants were excluded due to: (I) body mass index ≥ 30 kg/m^2^ (*n* = 6); (II) withdrawal from participation in the study (*n* = 13); (III) not performing the initial evaluations (*n* = 19); or other reasons (*n* = 3). Therefore, 59 participants were randomized into two groups, the HIIT group and the interval sprint training (SIT) group.

**Figure 1:**
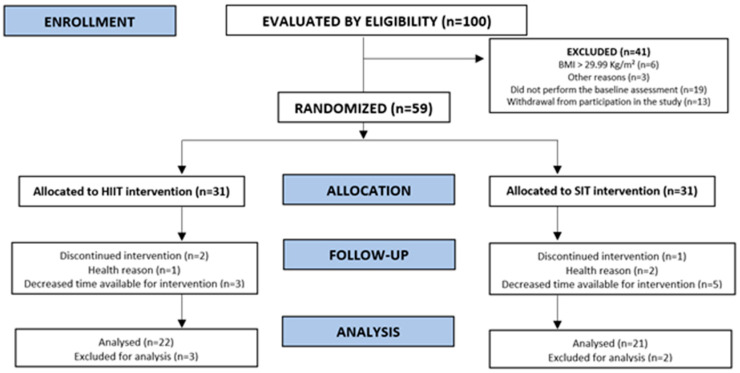
Flow diagram of participants.

Sixteen participants (~27%) abandoned the study for reasons comprising health problems (*n* = 2), alterations in time availability for practicing the training sessions (*n* = 9), and no reported reason (*n* = 5). (Figure 1) With this, the final sample consisted of 43 participants (HIIT, *n* = 22; and SIT, *n* = 21). The sample characteristics after allocation into groups at baseline are presented as mean and standard deviation (Table 1). All of the participants were informed about the objectives, procedures, possible discomforts, risks, and benefits of the study and signed the informed consent form. This study was derived from the Project Effect of different HIIT protocols in young people, submitted and approved by the Ethics Committee of the Federal University of Goias (approval number: 1,542,353), and is in accordance with the Declaration of Helsinki.

### 2.2. Study Design

**Figure 2:**
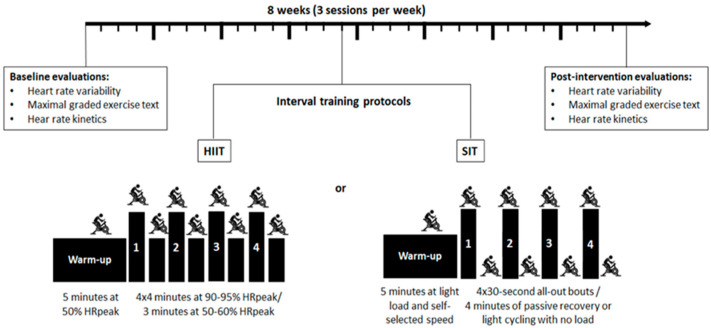
Study design. HIIT, high intensity interval training; SIT, sprint interval training. 1–4 effort sessions.

After the initial evaluations, the participants were randomized into two interval training groups: HIIT or SIT. The training protocols were performed three times a week (Monday, Wednesday, and Friday), totaling 24 training sessions over eight weeks; all sessions were held in the morning. One week before and after the intervention period, the participants were submitted to an HRV assessment and cardiorespiratory fitness assessment followed by HR kinetics assessment (Figure 2). During the study period, participants were asked to avoid any form of physical exercise beyond the study protocols.

### 2.3. Assessment of Cardiorespiratory Fitness

The cardiopulmonary test (TCP) was performed using a cycle ergometer with electromagnetic exercise brake with maximum classification (CG04, Inbramed, Sao Paulo, Brazil) and metabolic system (VO2000, MedGraphics, Saint Paul, United States of America coupled to the participant. TCP consisted of a two-minute warm-up at 50 W, followed by increments of 25 W every minute until exhaustion (failure to maintain a cadence above 50 rpm). Subsequently, two minutes of recovery were performed at 50 W and a cadence of 60 rpm. Oxygen volume (V-O2) (mL·kg^−1^·min^−1^) and HR (bpm) were continuously monitored. Peak V-O2 was defined as the highest mean value of V-O2 of 10 s with inclusion criteria consistent with conventional V-O2max (e.g., inability to sustain the workload in relation to HR > 95% of the predicted HR for age, respiratory exchange ratio in maximal exercise ≥ 1.1 and maximum perceived effort rating on the Borg scale 6–20) [26,27]. The heart rate peak (HRpeak) was defined as the highest HR achieved during the test.

### 2.4. R-R Record and Heart Rate Variability

The R-R intervals were recorded with the participant resting in a silent room, with controlled temperature (24 °C) and always between 8 a.m. and 12 p.m. to avoid variations in physiological responses due to circadian influences. Participants were instructed to have an empty bladder during the test, not to consume coffee and/or stimulants or alcoholic beverages in the previous 24 h, and not to perform any type of exercise for 48 h before the evaluation. In addition, participants were advised not to eat copious amounts of food in the two hours prior to the evaluation.

The R-R intervals were evaluated while the participant was at rest (sitting position) for six minutes in absolute silence, without sudden movements and with guidance to not fall asleep. The R-R intervals were collected by beat by a cardiofrequency meter (Polar^®^ model V800, Electro Oy, Helsinki, Finland) and a h10 coded transmitter placed on the participant’s chest at the height of the 5th intercostal space.

For HRV analyses, the zones of greatest signal stability were selected, containing 256 consecutive beats. The analyses were performed using linear and nonlinear models. For linear analysis of HRV in the time domain, the following indices were used: (I) square root of the mean quadratic differences between the adjacent normal R-R intervals (rMSSD), which reflects cardiac parasympathetic modulation; and (II) standard deviation of all normal R-R intervals recorded in a time interval (SDNN), which reflects the overall variability [28].

HRV analysis by geometric methods was performed by the indices obtained in the Poincaré plane: (I) dispersion points perpendicular to the identification line, which seems to be an instantaneous index of beat-to-beat variability (SD1); (II) scatter points along the identity line, which represents HRV in a long-term record (SD2); and (III) the ratio between short and long variations of R-R intervals (SD1/SD2). These indices express the complexity of HRV, which has a chaotic behavioral capacity, being highly capable of adapting to abrupt daily changes [28].

HRV analysis by nonlinear methods was performed via: (I) symbolic analyses, in which the R-R intervals (256 beats) are evenly distributed at 6 levels, with each beat receiving a symbol (from 0 to 5); then, sequences of 3 symbols (patterns) are constructed from the sequence of symbols and grouped into 4 families. The patterns without variation (0V), representing sympathetic cardiac modulation; patterns with a variation (1V), representing the vagal sympathetic baling; and patterns with two similar variations (2VS) or patterns with two different variations (2VD), both representing parasympathetic modulation; (II) the Shannon entropy (ES) index, calculated to provide complexity/form of distribution of patterns; and (III) Conditional Entropy (EC) that provides information on the organization of heartbeats and their predictability and lower complexity, ranging from 0 (null information, maximum predictability) to 1 (maximum information, minimum predictability) [29,30,31].

The HRV data for linear analyses in the time domain (R-R, SDNN, rMSSD) and nonlinear intervals (SD1, SD2, SD1/SD2 ratio) were obtained by Kubios^®^ HRV Analysis Software (version 3.0.2). The symbolic analysis, ES, and EC were obtained through the program of routine scans Scienze Precliniche, Universita’ degli Studi di Milano^®^, Milan, Italy [31].

### 2.5. Evaluation of Heart Rate Kinetics

A HR kinetics evaluation was performed during the transition from rest to exercise (FCon) and during the recovery period (HRoff) and was collected in TCP and subsequently filtered and analyzed with an ad hoc routine developed using OriginPro 8.0^®^ (OriginLab, Northampton, MA, USA). This algorithm applies an exponential model to the data corresponding to the full recovery period. A nonlinear algorithm that minimizes the sum of squared errors as a convergence criterion was used to determine the best parameters for the resulting exponential curve. The function was included only in the analysis if r > 0.95. Kinetics was modulated using the exponential time function, where “TAU” is time, “HRpeak” is the peak HR at the end of TCP, and “AMP” is the amplitude of HR reduction after the end of exercise.

The parameters of kinetic analysis (adaptation speed and amplitude in steady state) were calculated using a computer program created in LabVIEW^®^ (National instruments, Austin, TX, USA), following standard procedures. The quality of the adjustment was evaluated by the analysis of residues, correlation coefficients, and the 95% confidence interval range.

### 2.6. Delta Analysis

HR data were also characterized by analysis of HR deltas. The selected intervals of the recovery period (0–195 s, every 30 s) were subtracted from the HRpeak achieved in the TCP. The intervals were measured by calculating the mean HR in the 5 s before and after each moment. The larger the delta, the faster the HR adjustment and the better cardiac autonomic modulation through the adaptive capacity of the autonomic nervous system during exercise.

### 2.7. Intervention with Interval Training

During the study period, the participants were requested to avoid any form of physical activity besides the study protocols. The HIIT protocol consisted of a five-minute warm-up on a cycle ergometer (Evolution SR, Schwinn^®^, Chicago, IL, USA) at 50% of the HRpeak obtained in the TCP (FT1, Polar^®^, Helsinki, Finland), followed by four sessions of four-minute efforts between 90 and 95% of the CF interspersed with three minutes of active recovery in 50–60% of the HRPeak [32]. The load was adjusted when the HR deviated from the established zone. During recovery, the cadence was self-selected and the load was reduced to a minimum by one of the researchers. The SIT group performed a five-minute warm-up with light load and self-selected speed, followed by four sessions of 30 s in maximum effort, interspersed with four minutes of recovery (passive or light cycling without load) [33]. All training sessions for both groups were directly supervised by professionals with experience in prescribing training, in a proportion of one supervisor per volunteer, and standardized verbal stimuli were offered. The research team constantly monitored and questioned participants to verify that they followed the recommendations and recorded any adverse events (dizziness, nausea, muscle pain, etc.).

### 2.8. Statistical Analysis

The normality of the data was tested using the Shapiro-Wilk test, the homogeneity of variances was evaluated using the Levene test. An independent *t*-test was used to test general characteristics (age, height, body mass, and body mass index) between the HIIT and SIT groups at baseline. A 2 × 2 bidirectional variance analysis (ANOVA) was used to evaluate the effect of the interaction between the group (HIIT and SIT) × time (pre and post) for HRV indices (SDNN, rMSSD, HF, LF, HF/LF, SD1, SD2, SD1/SD2, ES, EC, 0V, 1V, 2VS, and 2VD) and HR kinetics parameters (onTAU, onAMP, offTAU, and offAMP). The differences between the groups were also evaluated by a covariance analysis (ANCOVA), considering the peak of V-O2 in the baseline as covariate. A 2 × 13 ANOVA was used to evaluate the effect of the interaction between the groups (HIIT and SIT) × moment (T0, T15, T30, T45 … T195 s) for HR after TCP performed at the beginning of the trial and after the intervention period. A 2 × 13 ANOVA was used to evaluate the effect of the interaction between time (pre and post) × moment (T0, T15, T30, T45 … T195 s) for HR in the HIIT and SIT groups. When necessary, the post-hoc test was performed by multiple comparisons using the least significant difference (LSD) correction. The significance level was established at 5%. All of the analyses were performed using IBM SPSS Statistics for Windows, version 20 (IBM Corp., Armonk, NY, USA).

## 3. Results

### 3.1. Participants

Forty-three women with a mean age of 29.96 ± 6.25 years participated in the analyses. The women had a BMI of 24.79 ± 3.30. Data from baseline measurements after group allocation did not show statistically significant results for weight or BMI variables. 

### 3.2. Heart Rate Variability and VO2

ANOVA 2 × 2 did not reveal significant group × time interaction for any of the variables studied. However, there was a significant group effect for VO2 (F [1.86] = 12.523; *p* = 0.001), indicating that, regardless of time, the HIIT group had higher VO2 than the SIT group. A significant effect of time (pre vs. post) was also observed for the variables VO2 (F [1.86] = 8.993; *p* = 0.004), RMSSD (F [1.86] = 10.465; *p* = 0.002), SDNN (F [1.86] = 9.434; *p* = 0.003), SD1 (F [1.86] = 9.512; *p* = 0.003), and SD2 (F [1.86] = 6.750; *p* = 0.011), indicating that, regardless of the group, interval training increased VO2 and HRV-related variables (Table 2). After adjustment for VO2peak, ANCOVA revealed that the effect of time for RMSSD (F [1.86] = 6.494; *p* = 0.013), SDNN (F [1.86] = 6.422; *p* = 0.013), SD1 (F [1.86] = 5.665; *p* = 0.020), and SD2 (F [1.86] = 4.841; *p* = 0.031) remained.

### 3.3. Entropy and Symbolic Analysis

ANOVA 2 × 2 showed significant group x time interaction for the variables ES (F [1.86] = 6.87; *p* = 0.011), EC (F [1.86] = 5.42; *p* = 0.023), 0V (F [1.86] = 3.91; *p* = 0.051), and 2LV (F [1.86] = 5.16; *p* = 0.026). The HIIT group presented better results in comparison with the SIT group for the variables 0V (*p* = 0.022), ES (*p* = 0.004), and EC (*p* = 0.025) at the post-protocol moment, demonstrating the best results for HRV complexity for the HIIT group (Table 2). 

### 3.4. Heart Rate Kinetics

In the evaluation of HR kinetics performed by deltas of the moments (0–195 s) of recovery, a 2 × 2 ANOVA was performed and a significant moment effect was found (F [1.86] = 40.374; *p* < 0.001), however, there was no effect of the group (F [1.86] = 2.960; *p* = 0.086) or moment × group interaction (F [1.86] = 0.438 *p* = 0.956) in the pre-time period for both groups. Meanwhile, a significant moment effect was also found in the post-time period (F [1.86] = 82.813; *p* < 0.001), alongside a significant group effect (F [1.86] = 8.003; *p* = 0.005), but there was no significance in relation to the group (F [1.86] = 2.960; *p* = 0.086) or moment × group (F [1.86] = 0.249 *p* = 0.997).

**Figure 3:**
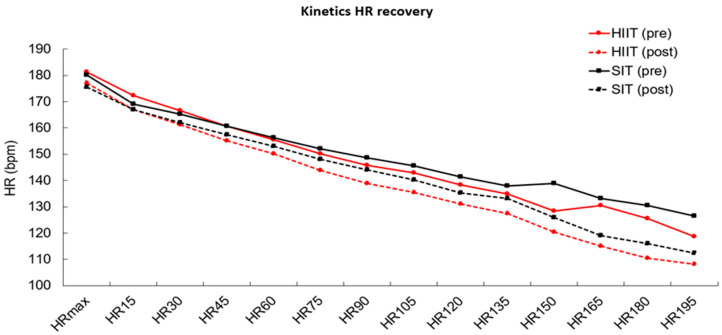
Kinetics of heart rate (HR) recovery after maximal graded exercise test before and after eight weeks of high intensity interval training (HIIT) or sprint interval training (SIT).

In the intragroup analysis, HIIT presented significance in relation to time (F [1.86] = 42.947; *p* < 0.001) and in relation to the moment (F [1.86] = 74.873; *p* < 0.001) but no time × moment interaction (F [1.86] = 0.617 *p* = 0.835) was identified. The same was demonstrated in the SIT group, in which a significant time effect was found (F [1.86] = 22.351; *p* < 0.001) as well as moment effect (F [1.86] = 41.194; *p* < 0.001); however, there was no time × moment (F [1.86] = 0.741 *p* = 0.723) (Figure 3).

## 4. Discussion

Several studies have investigated the influence of physical exercise on HRV; however, few have compared HRV responses following different high intensity training protocols. Our main finding was that eight weeks of interval training was able to produce positive effects on cardiac autonomic modulation in healthy women, with better results for HIIT over SIT in this population.

The best results are related to nonlinear indices, which are important parameters of cardiac autonomic modulation and contribute to the prevention of arrhythmias and sudden cardiac death [34,35]. In our study, this analysis presented significant results for the HIIT group, with an increase of 78.23% in the 2LV% index and an increase of 47.43% in the 0V% index, respectively, indicating parasympathetic and sympathetic modulation; in this case, the values express better cardiac autonomic adaptation and the possibility of reductions in resting HR, another index demonstrating cardiovascular health [8].

Several studies have shown that cardiac cycle oscillations throughout the day are a demonstration of good cardiac automation; these fluctuations of HR are essential as they ensure that the heart adapts to the needs of different levels of activity during the phases of exercise, rest, daily activities, and sleep, by decreasing or increasing cardiac output [3,36,37]. In our study, this adaptation was perceived both in the improvement of the sympathovagal balance represented by HRV indices and by the better behavior during the exercise recovery demonstrated by the analysis of HR kinetics by deltas, with no proven superiority between the protocols.

Significant results were found in relation to time for variables related to cardiac autonomic modulation, such as RMSSD, SDNN, SD1, SD2, and HR kinetics, and these demonstrated that there is an improvement in cardiac autonomic modulation by the practice of interval training, either via SIT or HIIT, with no difference between the protocols for these variables.

An increase in vagal modulation after high intensity training represents better adaptive capacity to exercise through changes at the central level. In our study, the performance of high intensity exercises for eight weeks presented results that differ from the previous literature, which has demonstrated no superiority between the groups of different types of protocols [38]. In our study, superiority was proven for the HIIT group by the nonlinear analysis of HRV, demonstrated by non-symbolic indices (0V, 2VD) and entropy (ES, EC).

In our study, HR kinetics presented a positive result, rapid recovery in both interval training groups; however, studies have shown the higher the intensity of exercise, the greater the interference in HRV recovery, justified by the amount of non-oxidizing energy and the large stimulation of the metaboreflex that generates late recovery due to a delay in parasympathetic activation [39,40]. As the exercise was interval training, it is likely that, in times of passive recovery, the interruption of the metaboreflex stimulus may have influenced the improvement of HR behavior.

The recovery phase after interruption of exercise is understood as the abrupt removal of the central command associated with decreased feedback from muscle mechanoreceptors, decreasing baroreflex action and, consequently, HR, which at this time is predominantly controlled by an increase in parasympathetic modulation. Therefore, the initial minute, described as the parasympathetic reactivation phase [41,42,43], was presented in our study by the evaluation of delta kinetics, demonstrating no superiority between the groups, although the SIT group did show a slight slowing of the parasympathetic increment, differentiating it from that presented by the studies [7,43] in which this reactivation was inversely proportional to the intensity and duration of the exercise and in this case, the protocol was of a lower intensity and shorter duration.

The superior results for HIIT in some parameters may be related to the duration of the effort. Previous studies have revealed that longer duration protocols, even if performed at a lower intensity, promote higher increases in oxygen consumption and heart rate, as well as more pronounced alterations in autonomic modulation [20]. It is likely that this higher acute stress also generates more chronic adaptations, due to the greater overload on the cardiovascular system [16].

### Limitations and Future Studies

The main limitation of our study was the abandonment of 27% of the population for various reasons, including change in city, family issues, and dental surgery. As future perspectives, it is suggested that controlled clinical trials be performed with probabilistic sampling to compare several training protocols in order to unveil the volume, intensity and types of exercises that present better results in relation to cardiac autonomic modulation. Here, we can clearly observe the good results obtained through the practice of interval training, since it is a healthy population, it is necessary to carry out further investigations in populations with cardiometabolic diseases to expand the benefits to the general population.

## 5. Conclusions

Eight weeks of interval training was able to produce benefits on cardiac autonomic modulation and cardiorespiratory conditioning in our study conducted with healthy women. It was possible to determine that the HIIT with the highest training volume was able to produce the best results related to the sympathovagal balance sheet, demonstrating this method to be an effective alternative for prescription medications.

## Figures and Tables

**Table 1:** Anthropometric characteristics of participants before interval training intervention.

	HIIT (*n* = 22)	SIT (*n* = 21)
Age (years)	31.1 ± 6.5	28.8 ± 6.0
Height (m)	164.04 ± 4.75	164.95 ± 4.91
Body mass (kg)	66.70 ± 10.16	67.86 ± 8.32
Body mass index (kg/m²)	24.60 ± 3.46	24.98 ± 3.19

HIIT, high intensity interval training; SIT, sprint interval training.

**Table 2:** Cardiorespiratory fitness, HRV indices and HR kinetics parameters at baseline and after the intervention period.

Variables	HIIT (*n* = 22)		SIT (*n* = 21)		Group	Time	Group × Time
	Pre	Post	Pre	Post			
$\dot{v}\dot{v}$O_2_peak (mL/kg/min)	37.3 ± 7.6	42.1 ± 6.0	32.0 ± 7.7	36.5 ± 7.0	0.001 *	0.004 *	0.924
**HRV índices**							
RMSSD (ms^2^)	29.7 ± 13.7	36.0 ± 20.8	24.7 ± 9.2	41.0 ± 18.6	0.994	0.002 *	0.159
SDNN (ms^2^)	34.3 ± 10.8	37.2 ± 13.5	29.4 ± 8.4	42.1 ± 13.5	0.990	0.003 *	0.059 *
SD1 (ms)	21.7 ± 10.1	25.5 ± 14.7	17.5 ± 6.5	29.1 ± 13.2	0.907	0.003 *	0.122
SD2 (ms)	43.2 ± 13.8	45.4 ± 14.6	37.5 ± 10.8	51.1 ± 16.2	0.998	0.011 *	0.062
SD2/SD1	2.3 ± 1.2	2.1 ± 0.7	2.3 ± 0.7	2.0 ± 0.6	0.692	0.133	0.826
**Entropy**							
ES	3.10 ± 0.8	3.81 ± 0.4	3.53 ± 0.75	3.64 ± 0.54	0.657	0.004 *	0.011 *
ECN	0.70 ± 0.10	0.77 ± 0.11	0.71 ± 0.11	0.75 ± 0.09	0.864	0.031 *	0.516
EC	0.92 ± 0.28	1.22 ± 0.19	1.06 ± 0.29	1.10 ± 0.22	0.887	0.003 *	0.023 *
**Simbolic Analisys**							
0V%	35.04 ± 20.10	18.42 ± 13.09	25.79 ± 19.49	26.37 ± 15.73	0.675	0.022 *	0.051 *
1V%	41.30 ± 9.38	44.57 ± 6.15	44.48 ± 8.58	45.03 ± 6.29	0.222	0.200	0.551
2LV%	7.81 ± 7.15	13.92 ± 6.51	14.18± 9.22	12.04 ± 7.92	0.146	0.193	0.026 *
2UV%	15.82 ± 8.89	23.05 ± 13.58	15.51 ± 8.81	16.53 ± 9.82	0.205	0.060	0.254

Legenda: HRV: heart rate variability; HR: heart rate; HIIT: high intensity interval training; SIT: sprint interval training; $\dot{v}\dot{v}$O_2_peak: peak oxygen uptake; SDNN: standard deviation of all normal RR intervals recorded in a time interval, expressed in milliseconds; rMSSD: square root of the mean squared differences between the adjacent normal RR intervals, in a time interval, expressed in milliseconds; SD1: instantaneous beat-to-beat variability index; SD2: long-term vfc record; SD1/SD2: ratio between SD1 and SD2; ES: Shannon entropy; EC: conditional entropy; 0V: patterns without variations; 1V: patterns with a variation; 2LV: patterns with two similar variations; 2UV: patterns with two different variations. * Statistical significance: *p* < 0.05

## Data Availability

The researchers can be contacted to make the data available.
